# Urinary exosome-derived microRNAs reflecting the changes of renal function and histopathology in dogs

**DOI:** 10.1038/srep40340

**Published:** 2017-01-11

**Authors:** Osamu Ichii, Hiroshi Ohta, Taro Horino, Teppei Nakamura, Marina Hosotani, Tatsuya Mizoguchi, Keitaro Morishita, Kensuke Nakamura, Yuki Hoshino, Satoshi Takagi, Noboru Sasaki, Mitsuyoshi Takiguchi, Ryo Sato, Kazuhisa Oyamada, Yasuhiro Kon

**Affiliations:** 1 Laboratory of Anatomy, Department of Biomedical Sciences, Graduate School of Veterinary Medicine, Hokkaido University, Kita 18, Nishi 9, Kita-ku, Sapporo 060-0818, Japan; 2 Laboratory of Veterinary Internal Medicine, Department of Veterinary Clinical Sciences, Graduate School of Veterinary Medicine, Hokkaido University, Kita 18, Nishi 9, Kita-ku, Sapporo 060-0818, Japan; 3 Department of Endocrinology, Metabolism and Nephrology, Kochi University School of Medicine, Kohasu, Oko-cho, Nankoku 783-8505, Japan; 4 Section of Biological Safety Research, Chitose Laboratory, Japan Food Research Laboratories, Bunkyo 2-3, Chitose 066-0052, Japan; 5 Veterinary Teaching Hospital, Graduate School of Veterinary Medicine, Hokkaido University, Kita 18, Nishi 9, Kita-ku, Sapporo 060-0818, Japan; 6 Matsubara Animal Hospital, Taijo 1-174-1, Matsubara 580-0044, Japan.

## Abstract

MicroRNAs act as post-transcriptional regulators, and urinary exosome (UExo)-derived microRNAs may be used as biomarkers. Herein, we screened for UExo-derived microRNAs reflecting kidney disease (KD) status in dogs. Examined dogs were divided into healthy kidney control (HC) and KD groups according to renal dysfunction. We confirmed the appearance of UExo having irregular globe-shapes in a dog by immunoblot detection of the exosome markers, TSG101 and CD9. Based on our previous data using KD model mice and the data obtained herein by next generation sequencing of UExo-derived microRNAs in dogs, miR-26a, miR-146a, miR-486, miR-21a, and miR-10a/b were selected as candidate microRNAs. In particular, UExo-derived miR-26a and miR-10a/b were significantly decreased in KD dogs, and miR-26a levels negatively correlated with deteriorated renal function compared to the other miRNAs. UExo-derived miR-21a levels corrected or not to that of internal control microRNAs in UExo, miR-26a and miR-191, significantly increased with renal dysfunction. In kidney tissues, the decrease of miR-26a and miR-10a/b in the glomerulus and miR-10b in the tubulointerstitium negatively correlated with deteriorated renal function and histopathology. Increased miR-21a in the tubulointerstitium rather than in the glomerulus correlated with deteriorated renal histopathology. In conclusion, microRNAs reflecting the changes in renal function and histopathology in dogs were identified in this study.

In clinical veterinary medicine, serum blood urea nitrogen (BUN) and creatinine (Cr) are universally used as renal function markers. In dogs and cats, serum Cr level is used to grade patients with chronic kidney disease (CKD) into several clinical stages proposed by the International Renal Interest Society for adequate therapeutic strategy (http://www.iris-kidney.com/guidelines/staging.html). Our previous findings suggest that CKD pathogenesis differs among companion animals, and the glomerulus (Glo) and tubulointerstitium (TI) tend to be injured in dogs and cats, respectively^1^. The elevation of serum Cr indicates renal dysfunction. However, it is difficult to estimate specific injured parts of the kidney from the results of serological analysis. Urinary protein and Cr are also useful markers for glomerular function and concentrating capacity, respectively. We recently focused on identifying a potential microRNA (miRNA) as a novel disease marker as well as therapeutic target in animal kidney diseases (KD)^2^,^3^,^4^.

miRNAs are stable small non-coding RNAs (20–25 bp) with high evolutionary sequence conservation that act as posttranscriptional regulators of target genes by binding to complementary sequences within specific target mRNAs. The close relationship between the progression of several diseases and miRNAs is well studied in both experimental rodents and humans^5^. In the kidneys, miR-21 participates in renal fibrosis by targeting transforming growth factor beta (TGF-β)/Smad signaling^6^. We also demonstrated that altered expression of miR-26a and miR-146a correlated with the progression of glomerular podocyte injury and tubulointerstitial inflammation in CKD model mice, respectively^2^,^3^. In veterinary medicine, although miRNA expression profile in companion animals is unknown, we recently identified miRNAs expressed in the renal cortex and medulla of dogs and cats by using next-generation sequencing (NGS)^4^.

miRNAs are also found in body fluids such as the serum, plasma, saliva, milk, and urine^7^. Urinary miRNA is stable because it is included in 40–100 nm nano-sized vesicles called “exosomes”^8^. Exosome is a type of extracellular vesicles such as microvesicles, apoptotic bodies, or ectosomes^9^. Exosomes are derived from the budding of endosomal membranes, resulting in the formation of multivesicular bodies (MVBs) and produced by the fusion of MVBs with the plasma membrane. Therefore, exosomal membrane molecules such as CD9, CD63, and CD81 and the proteins involved in exosomal biogenesis such as programmed cell death 6 interacting protein (also known as ALIX) or tumor susceptibility 101 (TSG101) are used as representative exosomal markers^10^.

Urinary exosome (UExo)-derived miRNAs may be novel diagnostic markers for KD. Solé *et al*. reported that miR-29c levels in UExos correlated with the chronicity and glomerular sclerosis in human patients with lupus nephritis^11^. Furthermore, miR-1915 and miR-663 levels in UExo were downregulated in patients with focal segmental glomerulosclerosis compared to healthy controls, whereas those of miR-155 were upregulated in these patients^12^. We also reported altered levels of miR-26a and miR-146a in the urine of CKD model mice^2^,^3^. Thus, changes of UExo-derived miRNAs significantly correlate with the progression of KD. However, there is no evidence in companion animals.

In the present study, by using dog UExo and NGS, we identified novel miRNA candidates reflecting changes of renal function or renal histopathology and compared their clinicopathological significance with KD-related miRNAs already identified in our previous studies.

## Results

### Appearance of UExo and UExo-derived RNAs in healthy dogs

**Figure 1:**
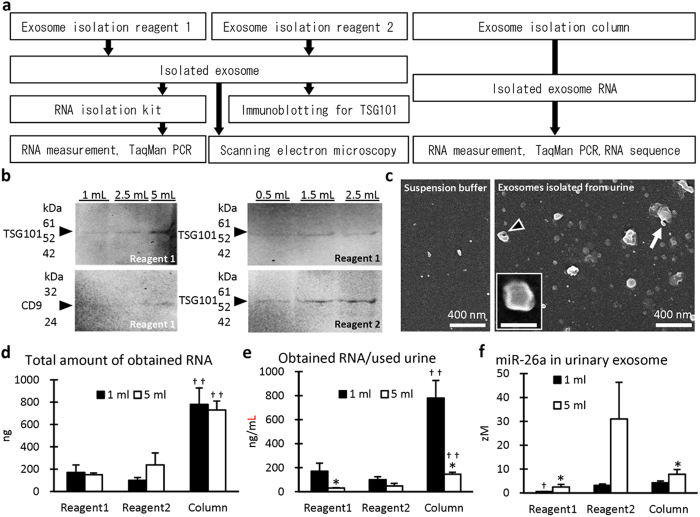
Analysis of urinary exosome-derived RNAs in dogs. (**a**) Flowchart of protocols used to analyze urinary exosome and exosome-derived RNAs in this study. (**b**) Detection of the urinary exosome markers, TSG101 and CD9, in urinary exosome-rich fractions. Reagent 1 or 2 was used in this experiment. “mL” at the top of lane indicates the volume of urine used. Arrowheads indicate the predicted bands for TSG101 or CD9. Fresh urine samples were used. (**c**) Ultrastructure of dog urinary exosome. Suspension buffer or re-suspended exosome rich fraction was mounted on a copper grid covered membrane and observed under scanning electron microscopy. The dog exosomes showed irregular round shapes and a diameter of about 50–200 nm. Some of them presented a pit (arrowhead) or process (arrow). Fresh urine samples were used. (**d**) Amount of RNA obtained from urinary exosome-rich fraction in dogs using 3 different methods. (**e**) Recovery efficiency of RNA obtained (obtained RNA/volume of urine used) from urinary exosome-rich fraction in dogs using 3 different methods. (**f**) miRNA level in RNA obtained from urinary exosome-rich fraction by 3 different methods in dogs. miR-26a was analyzed as a representative miRNA abundantly expressed in the animal kidney. TaqMan PCR method. For graphs d-f, four pooled samples of frozen urine obtained from more than 10 dogs for each sample were used in this experiment. Values = mean ± SE. *Significant difference between 1 and 5 mL groups analyzed by Mann-Whitney *U* test (*P* < 0.05). ^†,††^Significant difference from the other method in same urine volume group analyzed by Scheffé method (*P* < 0.05, *P* < 0.01, respectively) following Kruskal-Wallis test.

Figure 1a summarizes the analysis protocols of UExos and UExo-derived total RNAs. In Fig. 1b and c, UExos collected by reagent 1- or 2-based method from fresh urine from a dog, showing normal kidney function, were examined. For immunoblotting, bands corresponding to the exosome markers, TSG101 and CD9, were detected (Fig. 1b), but the obvious band for the latter was detected when UExos were collected from 5 mL of urine. Furthermore, TSG101-derived band was detected in both reagent 1- and 2-based UExo isolation protocols. Scanning electron microscopy (SEM) showed that dog UExos presented an irregular globe-shape with a diameter of approximately 50–200 nm, and some of them presented a pit or process (Fig. 1c).

Next, we examined the appearance of UExo and measured the amount of UExo-derived RNAs in a dog frozen urine samples by 3 different protocols. In Fig. 1d–f 4 pooled samples, collected from more than 10 dogs showing normal kidney function, were analyzed. The highest total amount of UExo-derived total RNAs was obtained by using the column method when compared with the other methods using both 1 and 5 mL of urine (Fig. 1d). In term of recovery efficiency of UExo-derived total RNAs (Fig. 1e), UExo-derived total RNAs tended to decrease with the volume of urine used in all examined protocols, and significant differences between the groups using 1 and 5 mL were detected in the reagent 1 and column method. Furthermore, the highest amount of UExo-derived total RNAs was obtained by using the column method when compared with the other methods in both groups using 1 and 5 mL of urine. Figure 1f shows the levels of miR-26a, which was reported as the miRNA abundantly expressed in the kidneys of mice, dogs, and cats^3^,^4^, in the obtained UExo-derived total RNAs. The miR-26a level tended to increase with the urine volume used in all examined protocols, and significant differences between the groups using 1 and 5 mL were detected in reagent 1 and column method. Thus, our data showed that UExo are detected in dogs and suggested that the amount of obtained UExo-derived total RNAs is not necessarily similar to that of miRNAs.

### UExo-derived miRNAs in healthy kidney control (HC) and KD dogs

**Figure 2:**
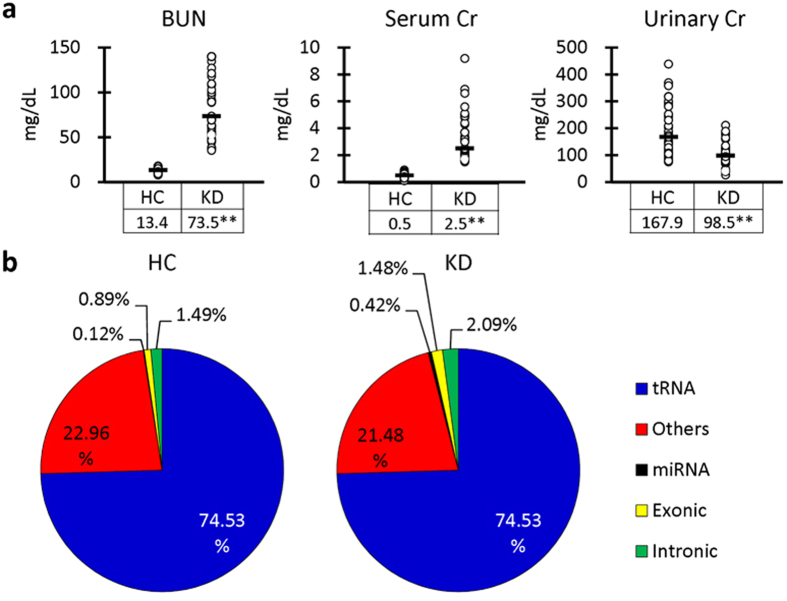
Analysis of renal function and urinary exosome-derived RNA sequences in dogs. (**a**) Clinical parameters of dogs grouped into healthy kidney control (HC, n = 37) and kidney disease (KD, n = 47). BUN: blood urea nitrogen. Cr: creatinine. Bars in graphs and values in tables show the median of each group. **Significant difference between HC and KD analyzed by Mann-Whitney *U* test (*P* < 0.01). (**b**) Percentage of each RNA obtained from urinary exosome-derived RNA in dogs by next generation sequencing. Pooled samples of healthy kidney control (HC, n = 37) and kidney disease (KD, n = 47) were analyzed. tRNA: transfer RNA.

Based on the serum Cr and BUN, urine color, and the diagnosis of a clinical veterinarian, the obtained urine samples were divided into 2 groups; HC (n = 37, median = 10-years-old) and KD (n = 47, median = 12-years-old). Clinical information of the examined dogs, including sex, age, and species is summarized in supplemental Table 1 and supplemental Fig. 1. Figure 2a shows the renal functions of HC and KD dogs. KD dogs showed significantly higher values in serum BUN and Cr and lower values in urinary Cr compared to HC dogs. NGS was performed by using these samples (Fig. 2b). Because of alignment, the percentage of UExo-derived miRNAs obtained were 0.12% and 0.42% in UExo-derived total RNAs of HC and KD dogs, respectively. Characteristically, most read sequences were derived from that of transfer RNA. As a result, 2,035 miRNAs were annotated.

Table 1 summarizes the results of UExo-derived miRNAs levels in HC and KD dogs. To refine the candidates, miRNAs showing more than 100 read numbers in both groups and a 2-fold difference (higher or lower) in KD dogs compared to HC dogs were selected. miR-3107, miR-486a, miR-21a, miR-10a, and miR-10b satisfied these requirements. miR-3107-5p was recently updated as miR-486b-5p, presenting the same mature sequence “uccuguacugagcugccccgag” as miR-486a-5p according to the mouse database (miRBase, http://www.mirbase.org/). Therefore, miR-3107 and miR-486a are essentially the same mature miRNAs. Furthermore, to identify an internal control miRNA in dog UExo, miRNAs showing more than 300 read numbers in both groups and less than a 2-fold difference (higher or lower) in the KD group compared to the HC group were selected. Among the selected miRNAs, we used miR-191 as an internal control in the subsequent study because it showed the lowest fold change between the HC and KD groups and was abundantly expressed in the kidneys of mice, dogs, and cats^3^,^4^.

### Levels of selected UExo-derived miRNAs in HC and KD dogs

**Figure 3:**
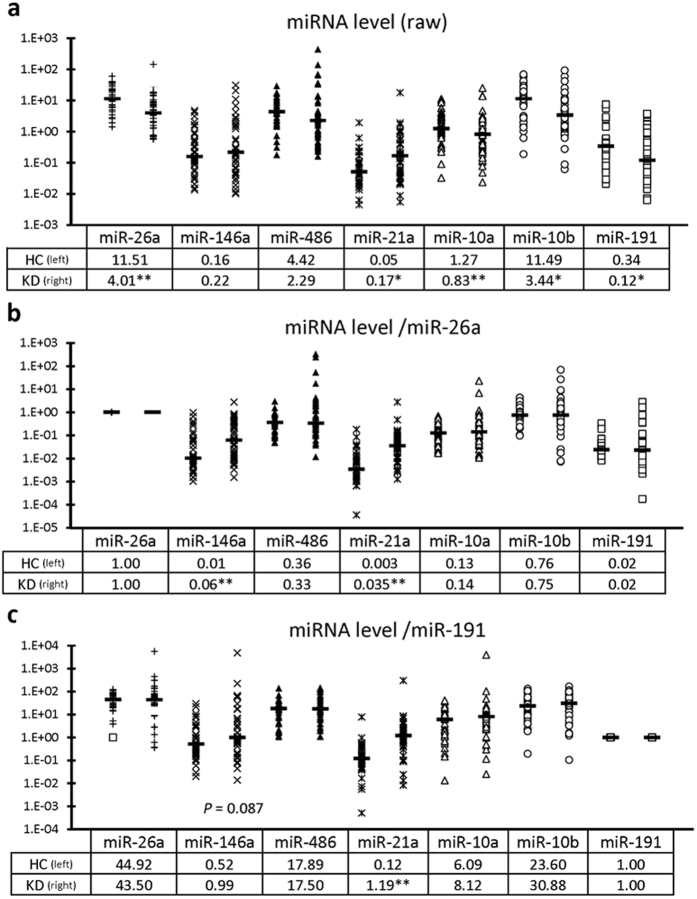
Level of urinary exosome-derived miRNAs in dogs. (**a**) Raw miRNA levels in urinary exosome-derived miRNA in dogs. (**b**) miRNA levels corrected to miR-26a level in urinary exosome-derived miRNA in dogs. (**c**) miRNA levels corrected to miR-191 level in urinary exosome-derived miRNA in dogs. Dogs were divided into healthy kidney control (HC, n = 37) and kidney disease (KD, n = 47) groups. *, **Significant difference between HC and KD groups analyzed by Mann-Whitney *U* test (*P* < 0.05, *P* < 0.01, respectively). TaqMan PCR method. Bars in graphs and values in Tables show the median of each group.

Next, levels of selected miRNAs in UExo were quantified from the results presented in Table 1. Levels of miR-26a and miR-146a, which were differentially expressed in the kidneys of CKD mice compared to that in healthy control mice in our previous studies, were also quantified^2^,^3^. The raw levels of miR-26a, miR-10a, miR-10b, and miR-191 were significantly lower in UExo from KD dogs compared to those in UExo of HC dogs (Fig. 3a). In contrast, miR-146a and miR-21a levels tended to be higher in UExo of KD dogs compared to those in UExo of HC dogs. A significant difference was only detected in miR-21a levels between the groups. Additionally, we corrected their levels to the raw levels of miR-26a and miR-191 (Fig. 3b and c), which were selected as internal controls in dog UExo, based on their abundant expression in the kidneys of dogs^3^,^4^. The results are presented in Table 1. When the expression levels were corrected to that of miR-26a, the levels of miR-146a and miR-21a were significantly higher in UExo of KD dogs than that in UExo of HC dogs (Fig. 3b). When the expression levels were corrected to that of miR-191, the levels of miR-146a, miR-21a, miR-10a, and miR-10b tended to be higher in UExo of KD dogs than in UExo of HC dogs. A significant difference was detected in miR-21a levels between the groups (Fig. 3c). Furthermore, we examined the raw levels and the corrected levels of miRNAs in each sex group, including male, castrated male, female, and spayed female, and there was no significant sex-related difference (*P* > 0.05) in any miRNA as determined by Kruskal–Wallis test.

### Correlation between levels of UExo-derived miRNAs and renal function in HC and KD dogs

A significant positive correlation was detected between age and miR-146a (Table 2). Serum BUN was significantly and negatively correlated with miR-26a and miR-10b. Serum Cr was significantly and negatively correlated with miR-26a, miR-10a, miR-10b, and miR-191 and positively correlated with miR-21a. Urinary Cr was significantly and positively correlated with miR-26a, miR-486, miR-10a, miR-10b, and miR-191. When miRNA expression levels were corrected to that of miR-26a, age was positively correlated with miR-146a, and serum BUN and Cr were positively correlated with miR-146a and miR-21a, while urinary Cr was negatively correlated with these miRNAs. When the expression levels were corrected to that of miR-191, miR-21a was positively correlated with age, serum BUN, and Cr, while it was negatively correlated with urinary Cr.

### Renal expression of miRNAs in the kidneys of HC and KD dogs

**Figure 4:**
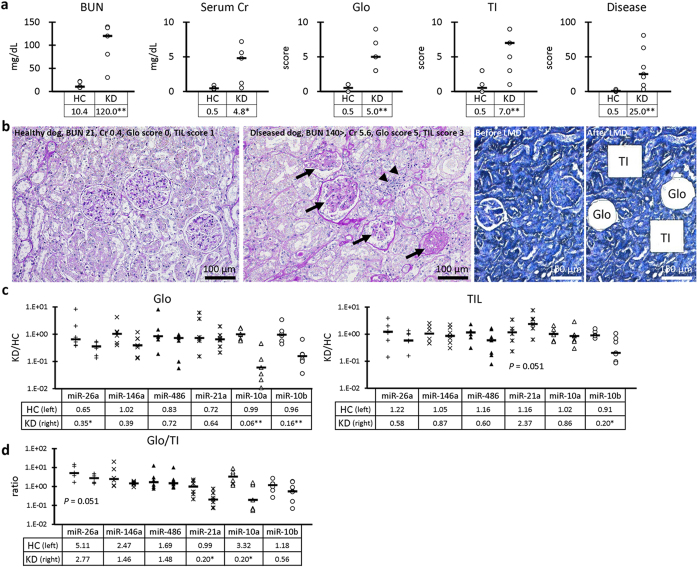
Level of miRNAs in the kidney of dogs. (**a**) Clinical and histopathological parameters of the analyzed dogs. Dogs were divided into healthy kidney control (HC) and kidney disease (KD) groups. *, **Significant difference between HC and KD groups analyzed by Mann-Whitney *U* test (*P* < 0.05, *P* < 0.01, respectively). Bars in graphs and values in tables show the median of each group. (**b**) Representative histopathological features of the analyzed dogs. In KD dogs, damages are observed in the glomerulus (Glo, arrows) and tubulointerstitium (TI, arrowheads). The histopathological scores of the Glo and TI as well as the serum values of blood urea nitrogen (BUN) and creatinine (Cr) are shown. Sections were stained with Periodic acid Schiff. Glo and TI containing areas were isolated by laser-microdissection (LMD) after toluidine blue staining. (**c**) miRNA expression in the Glo and TI collected by LMD was analyzed by using the TaqMan PCR method. Values are expressed as fold increase of KD compared to HC. (d)Glo to TI miRNA expression ratios. HC: n = 6. KD: n = 7. *, **Significant difference between HC and KD analyzed by Mann-Whitney *U* test (*P* < 0.05, *P* < 0.01, respectively). Data indicates the mean value of each column. Bars in graphs and values in the Tables show the median of each group.

Figure 4a presents renal functions and kidney damage scores of the dogs histopathologically divided into HC (n = 6, median = 11 years old) and KD (n = 7, median = 10.5 years old). Clinical information of the examined dogs is summarized in supplemental Table 2. Dogs with KD showed significantly higher values of serum BUN and Cr and damage scores for Glo, TI, and KD. Representative histopathological features of the kidneys of HC and KD dogs are shown in Fig. 4b. In KD dogs, the kidneys showed clear Glo and TI damages. Figure 4c shows local expression of miRNAs in the kidneys analyzed by laser-microdissection (LMD). The expression of all examined miRNAs was lower in KD compared to HC collected Glo. Significant differences were detected in miR-26a, miR-10a, and miR-10b between the groups. For collected TI, the expression of miR-21a tended to be higher in KD compared to HC specimens. The expression of other miRNAs tended to be lower. A significant difference was detected in miR-10b levels between the groups. We also calculated the miRNA expression ratio in Glo compared to TI (Fig. 4d). For the HC group, miR-26a, miR-146a, and miR-486 presented a Glo to TI ratio above 1.5, meaning that they were abundantly expressed in Glo rather than in TI, but miR-21a and miR-10b showed a 0.99 and 1.18 ratio, respectively, meaning that they were comparatively expressed in both Glo and TI. All parameters tended to be lower in KD dogs compared to HC dogs. Significant differences were observed in terms of miR-21a and miR-10a levels between the groups. Furthermore, the levels of miR-21a, miR-10a, and miR-10b were less than 1.0 in dogs with KD, meaning that they tended to be expressed in TI rather than in Glo in dogs with KD.

### Correlation between miRNA levels in the kidney and renal function in HC and KD dogs

For miRNA levels in the kidney tissues collected by LMD (Table 3), age did not correlate with the examined miRNA levels. Serum BUN and Cr, and Glo damage score were negatively correlated with Glo levels of miR-26a, miR-10a, and miR-10b. Serum Cr also negatively correlated with miR-146a levels. Both TI damage score and KD score were negatively correlated with Glo levels of miR-486, miR-10a, and miR-10b. Furthermore, serum BUN and Cr as well as Glo and TI damage scores, and KD score were negatively correlated with TI levels of miR-10b. Glo damage and KD scores were also negatively correlated with miR-21a levels.

Regarding the Glo to TI ratio (Table 4), miR-26a, miR-146a, and miR-10a were negatively correlated with Glo damage and KD scores, and miR-146a and miR-10a were negatively correlated with TI damage score and serum Cr, respectively. miR-10b was negatively correlated with age.

## Discussion

We examined the levels of UExo-derived miRNAs selected from both NGS and our previous reports^2^,^3^. Raw levels of UExo-derived miRNAs (Fig. 3a) can indicate 2 possibilities; 1) the quantitative changes of total amount of UExo or 2) the quantitative change of miRNAs in UExo. Because it is difficult to accurately measure the amount of UExo in dogs due to the lack of appropriate measurement techniques, we considered that internal control UExo-derived miRNAs were needed as an alternative. Therefore, we used miR-26a and miR-191 as internal controls, because they are known to be abundantly expressed in the kidneys of mice, dogs, and cats^3^,^4^. Additionally, miR-191 did not show drastic quantitative changes in the urine of HC and KD dogs in NGS (Table 1). miR-26a and miR-191 significantly decreased in UExo from KD dogs compared to that in HC dogs, suggesting that the total UExo amount tends to decrease in KD compared to HC. Therefore, its decreased level would also relate to a decrease in the total UExo amount in KD dogs. Furthermore, the raw levels of miR-26a and miR-191 in UExo correlated with changes of renal function indices such as serum BUN, serum Cr, or urinary Cr. Therefore, decreased UExo in KD might indicate altered renal function in the dog kidneys.

miR-10b also significantly decreased in KD dogs compared to HC dogs, and their levels closely correlated with renal function compared to the other miRNAs. Although differences in miR-10b and miR-10a levels did not match between TaqMan PCR and NGS, we analyzed these miRNAs because their read number was high (Table 1), and they are highly expressed in human urine^13^ as well as in dog and cat kidneys^4^. This methodological difference might be affected by the differences between individual samples in TaqMan PCR and pooled samples in NGS. On the other hand, the expression of UExo-derived miR-21a significantly increased in KD dogs compared to that in HC dogs. This increase was still observed after correction of the expression levels to those of internal control candidates such as miR-26a or miR-191 and closely correlated with changes in renal function indices. Thus, the amount of miR-21a in UExo, rather than raw levels of urinary miR-21a, increased during KD and correlated with changes in renal function in dogs.

Overall, the expression of all examined miRNAs tended to decrease in the Glo of KD dogs compared to that in HC dogs, but these changes were milder in TI than in Glo in KD dogs (Fig. 4c). We previously reported that Glo lesions in CKD such as podocyte injuries were more strongly correlated with renal dysfunction in dogs than in cats^1^. Furthermore, approximately 52% of renal biopsy samples from diseased dogs presented Glo injuries^14^. On the other hand, TI damages, including cell infiltration, fibrosis, polycystic lesions, and necrosis, were commonly observed in cat KD^15^,^16^. Therefore, the decreased expression of the examined miRNAs in Glo might be associated with the pathological characteristics of dog kidneys such as Glo-dominant pathogenesis in this species.

Decreased Glo miRNAs in KD such as miR-26a, miR-10a, and miR-10b significantly and strongly correlated with renal dysfunction and Glo injuries in dogs. These miRNAs also showed decreased levels in UExo and significant correlations with several parameters of renal dysfunction (Table 2). These 3 miRNAs are abundantly expressed in the mouse Glo. In particular, podocytes seemed to express miR-26a according to our mouse study^3^. As shown by the Glo/TI ratio (Fig. 4d), miR-26a and miR-10a also seemed to be expressed in the dog Glo compared to TI. Although there is no report about miR-10a in Glo cells, downregulation of miR-26a and miR-10a in Glo was reported in a CKD mouse model^3^. Decreased miR-26a in the kidneys is also demonstrated in diabetic nephropathy of humans and mice^17^. In humans, miR-26a levels decreased in the kidneys and urine of patients with lupus nephritis^18^. miR-26a silencing affected the cytoskeleton and differentiation of cultured mouse podocytes via altered expression of the actin family and vimentin^3^. Moreover, miR-26a downregulation is involved in the progression of diabetic nephropathy both in humans and in mice through enhanced TGF-β/connective tissue growth factor signaling^17^. Podocytes and mesangial cells can produce exosomes^2^,^19^,^20^. Thus, decreased miRNA expression in Glo, at least for miR-26a, would be a crucial pathological event in dog KD and correlates with decreased UExo due to altered exosome production or producing cell numbers in the Glo.

miR-10b decreased in both the Glo and TI of KD dogs, and this change was significantly correlated with renal dysfunctions. miR-10b is expressed in the kidneys of mice, dogs, and cats^3^,^4^. The role of miR-10b in KD is unknown, but a previous report suggested that upregulation of cAMP responsive element binding protein 1 by loss of miR-10b plays an important role in the tumorigenesis of renal cell carcinoma^21^. Furthermore, miR-10b is significantly downregulated in rejected allografts, and miR-10b inhibition in human endothelial cells recapitulated apoptosis, release of pro-inflammatory cytokines/chemokine, and chemotaxis of macrophages^22^. Based on the Glo/TI ratio of miR-10b (0.8 in KD *vs*. 1.2 in HC), miR-10b was expressed in both Glo and TI, but this ratio decreased with aging. Furthermore, miR-10b level decreased in UExo in KD dogs. Therefore, decreased miR-10b in both the kidney and UExo might reflect the abnormal characteristics of cells composing dog kidneys and/or the inflammatory status. Aging also affects these processes.

The levels of miR-486 in Glo and the Glo/TI ratio of miR-146a also significantly correlated with renal histopathological scores, although no significant change in both Glo and TI was detected between HC and KD dogs. For these miRNAs, although UExo-derived miR-146a/miR-26a ratio correlated with renal function parameters (Table 2), a constant tendency was not observed between the expression of miR146a and miR-486 in UExo and renal functional parameters. Age seemed to affect miR-146a levels in UExo. Recent studies reported that miR-486 in urinary sediments derived from urinary erythrocytes, not from renal parenchymal cells, significantly increased in human patients with IgA nephropathy^23^ and that miR-146a expression in the kidneys was specifically associated with inflammatory cell infiltration in CKD mice^2^. In the dog kidneys, the other miRNAs rather than miR-486 and miR-146a more closely correlated with renal pathogenesis or changes in renal functions.

miR-21a expression in the kidney is relatively low in mouse, dogs, and cats^3^,^4^. In the present study, miR-21a expression in TI tended to increase in KD, and Glo/TI ratio decreased in KD compared to HC dogs (0.20 in KD *vs*. 0.99 in HC), suggesting that gene expression and/or the number of cells expressing miR-21a in TI tended to increase in KD, but no significant correlation was observed between renal miR-21a expression and all examined renal functional parameters. Many studies indicated that miR-21a is upregulated in several animal models and humans suffering from KD, and is considered a key mediator of renal fibrosis^24^,^25^. miR-21a suppresses the expression of genes involved in mitochondrial and peroxisomal functions and the generation of reactive oxygen species in renal tubular cells and presents mitochondrial suppressive function, enhancing matrix deposition and nuclear factor kappa B signaling in fibroblasts^26^. In the present study, miR-21a expression in TI significantly correlated with Glo damage score rather than TI damage score, and this result might reflect that dog renal pathology tended to show Glo damage, as shown by the significant decrease of miR-26a in Glo of KD dogs. Otherwise, the time difference between miR-21a expression and histopathological manifestation of TI damage might explain the contradiction in the relationship between miR-21a expression in TI, Glo damage, and TI damage. Interestingly, miR-21a was upregulated in dog UExo (Fig. 3). Therefore, miR-21a would be an indicator of kidney tissue injuries independent of renal dysfunction in dog kidneys.

We extracted the UExo-rich fraction and subsequently obtained total RNA, including miRNAs (Fig. 1). The recovery efficiency of UExo-derived total RNA decreased with the increase of urine volume used by all examined methods, meaning that unknown inhibiting factors affect UExo-derived RNA extraction processes. However, the level of miRNAs (miR-26a was examined in this study) did not correlate with that of total RNA in UExo. As shown by immunoblotting, the band intensity of the exosomal marker, TSG101, seemed to increase with the urine volume used. Therefore, the recovery efficiency of UExo-derived miRNAs would increase with the urine volume used, while other coding or non-coding RNAs such as ribosomal, transfer, nuclear, and their degraded RNAs derived from urine supernatant or the extracellular vesicles except for exosomes such as microvesicles, apoptotic bodies, or ectosomes might interfere with the extraction of UExo-derived total RNA. Indeed, in our NGS using UExo-derived total RNAs extracted by the column method, the majority of read sequences partially derived from transfer RNAs (Fig. 2b). A previous report also showed that different RNA extraction methods affected the results of NGS of human UExo-derived miRNAs^13^. In this study, because the obtained sequences of UExo-derived miRNAs ranged from 0.12 to 0.42% in dog UExo-derived total RNAs, further modified methodology to specifically concentrate miRNA-derived sequences would lead to a more accurate UExo-derived miRNA analysis. Furthermore, the availability of good antibodies to detect markers of dog exosomes is limited although good antibodies for the detection of markers of human and mouse exosomes are readily available. The identification of appropriate protein markers for dog exosomes and the development of the corresponding antibodies are needed for future studies. In addition, all urine samples were obtained from animal hospitals and involved different breeds of dogs in both groups in this study, which may affect the obtained results. Therefore, the evaluation with the fixed constant method based on the appropriate method for UExo-derived miRNA extraction using high-affinity antibodies for UExo and in each dog breed would be important for UExo-derived miRNAs in clinical applications for humans as well as in veterinary medicine.

In conclusion, we identified several candidate miRNAs associated with altered renal functions and kidney tissue injuries from the miRNA expression data of UExos and kidney samples in dogs. In particular, based on our present data and several previous studies, miR-26a and miR-21a would be strong candidates indicating Glo and TI damages, respectively. Further studies of expression data of these miRNAs in UExo and kidney tissues are warranted for the application of UExo-derived miRNAs to clinical veterinary medicine.

## Methods

### Ethics statement

The investigators adhered to the Guide for the Care and Use of Laboratory Animals of Hokkaido University, Graduate School of Veterinary Medicine (approved by the Association for the Assessment and Accreditation of Laboratory Animal Care International). All sampling processes were carried out as part of clinical examination or diagnosis and the owners of the animals provided an informed consent. The present study retrospectively analyzed these collected samples for 2012 until 2016.

### Animal patients

The dog urine samples were obtained from patients in Veterinary Teaching Hospital, Hokkaido University (Sapporo, Japan) and Matsubara Animal Hospital (Osaka, Japan), and analyzed retrospectively. First, dogs showing serum Cr levels over 1.5 mg/dL were selected as candidates having a risk of KD (International Renal Interest Society; http://iris-kidney.com/guidelines/grading.html). Forty-seven dogs were diagnosed with KD based on the serum Cr as well as serum BUN, urine color, the diagnosis of a clinical veterinarian, and medical history from health record on the date of urine collection. KD samples showed a range of serum BUN (35.4–140.0 mg/dL) and Cr (1.5–9.2 mg/dL). HC samples were defined as samples from individuals having normal range of serum BUN (8.0–18.0 mg/dL) and Cr (0.1–0.9 mg/dL). All urine samples were stored at −3 0 °C until used.

For kidney tissues, samples were obtained at Hokkaido University when dogs were euthanized and autopsied. Firstly, the kidney samples showing normal renal histology and obvious renal lesions were divided into the HC and KD groups, respectively. The candidates showing elevated range of serum BUN (30.4–140.0 mg/dL) and Cr (0.5–7.2 mg/dL) were diagnosed as KD (n = 7). The candidates showing a normal range of serum BUN (8.3–21.8 mg/dL) and Cr (0.3–0.9 mg/dL) were diagnosed as HC (n = 6).

### Isolation of UExo-rich fraction

All urine samples (1 mL or 5 mL) were centrifuged at 2,000 × *g* for 30 minutes at 4 °C to remove cells and debris. From the urine supernatant, UExo-rich fraction was isolated by using Total Exosome Isolation (from urine) (reagent 1; ThermoFisher Scientific, Waltham, MA, USA) or miRCURY Exosome Isolation Kit-Cells, urine, and CSF (reagent 2; Exiqon, Vedbaek, Denmark) according to the manufacturer’s instructions. Briefly, for reagent 1, equal volumes of urine and reagent were mixed and incubated at room temperature for 1 hour. After incubation, the sample was centrifuged at 10,000 × *g* for 1 hour at 4 °C. For reagent 2, urine and reagent (2.5 to 1.0 ratio) were mixed and incubated at 4 °C for 1 hour. After incubation, the sample was centrifuged at 10,000 × *g* for 30 min at 20 °C. In both protocols, after aspiration of the supernatant, a pellet containing UExo was obtained and used for immunoblotting, SEM, or RNA analysis.

### Immunoblotting

Soluble proteins were extracted from isolated UExo-rich fraction using RIPA lysis buffer (Santa Cruz Biotechnology; Dallas, TX, USA). Immunoblotting was performed using the NuPAGE electrophoresis system (Life Technologies, Carlsbad, CA, USA) with the anti-TSG101 rabbit antibody (reactivity with human, mouse, rat, cow, dog, horse, and rabbit; Biorbyt, Cambridge, UK), the anti-CD9 rabbit antibody (reactivity with human, mouse, rat; Abcam, Cambridge, UK), and donkey anti-Rabbit IgG (H + L) secondary Alexa Fluor 488 conjugated antibody (ThermoFisher Scientific). Immune complexes were detected using Typhoon Variable-Mode Imager (GE Healthcare; Little Chalfont, UK).

### SEM

UExo-rich fraction isolated by using reagent 2 was suspended in 100 μL of Resuspension Buffer (Exiqon) containing 2% paraformaldehyde for 10 min. After fixation, 10 μL of sample solution was mounted on the Parafilm (Bemis, WI, USA) and covered with a grid (200 mesh) coated with excel support film (Nisshin EM, Tokyo, Japan) for 10 min. After washing with phosphate buffered saline (PBS) for 30 s twice, sample-attached grids were fixed with 2.5% glutaraldehyde for 5 min. After washing in distilled water (DW) for 30 s 7 times, samples were post-fixed with 1% osmium tetroxide for 30 min. After washing with DW for 30 s 7 times, samples were incubated with 10% samarium for 10 min. After washing with DW for 30 s 7 times, samples attached to the grid were mounted on the aluminum specimen stage and lightly sputter-coated with E-1030 (Hitachi, Tokyo, Japan). The specimen was observed on an S-4100 SEM (Hitachi) under the condition of 5 kV, 10 μA, and SE(U) mode.

### RNA purification from UExo-rich fraction

From the isolated UExo-rich fraction, total RNA was purified by using miRNeasy Micro Kit (Qiagen, Venlo, Netherlands). Total RNA from UExo was also obtained by using 1 step column kit (Urine Exosome RNA Isolation Kit; Norgen, Thorold, ON, Canada) according to the manufacturer’s instruction. All RNA solutions were adjusted to 100 μL with RNase-free water. The obtained RNA concentration was measured by NanoDrop 2000 (ThermoFisher Scientific).

### NGS

NGS was carried out as previously reported^4^. The quality of total RNA in isolated UExo-derived total RNAs obtained from the column-based method (urine 1 mL) was checked by using a Bioanalyzer (Agilent; Santa Clara, CA, USA), and samples from HC and KD animals were pooled as one sample for each group. Libraries were prepared using a TruSeq Small Library Preparation Kit (Illumina; San Diego, CA, USA) according to the manufacturer’s protocols and sequenced using 50-base reads acquired by using a HiSeq 2000 platform. The details of the sequence analysis of small RNAs targeting miRNAs are described in Supplemental Methods. The December 2011 (GRCm38/mm10) mouse (*Mus musculus*) genome data were used as reference (https://genome.ucsc.edu/).

### Histopathological examination and LMD

The kidneys were fixed using 10% neutral buffered formalin or 4% paraformaldehyde and were embedded in paraffin. To assess the severity of Glo damage, over 20 glomeruli per kidney were examined by using Periodic acid-Schiff (PAS)-stained sections. Each kidney sample was comprehensively scored according to the following criteria: score 0, no recognizable Glo lesion; score 3, a little PAS-positive deposition, mild proliferative or membranous lesions, mild glomerular hypertrophy; score 5, segmental or global PAS-positive deposition, proliferative or membranous lesions, and/or glomerular hypertrophy; score 7, the same as score 5 with PAS-positive deposition in 50% of regions of Glo and/or sever glomerular atrophy or hypertrophy; score 9, disappearance of capillary or capsular lumina, global deposition of PAS-positive material, periglomerular infiltration of inflammatory cells, and/or sever glomerular sclerosis. Similarly, to assess the severity of TI damage, over 20 regions in the renal cortex were examined at a 200-fold magnification. Each kidney sample was comprehensively scored according to the following criteria: score 0, no recognizable lesion in TI; score 3, a little PAS-positive materials in the tubular lumen, mild peritubular cell infiltration, mild hypertrophy of tubular basement membrane, and/or mild dilation of tubular lumen; score 5, several PAS-positive materials in the tubular lumen, peritubular cell infiltration, hypertrophy of tubular basement membrane, and/or dilation of tubular lumen; score 7, the same as score 5 with fibrotic feature in TI; and score 9, numerous PAS-positive materials in the tubular lumen, severe peritubular cell infiltration, hypertrophy of tubular basement membrane, atrophy of tubules, severe fibrotic feature, and/or severe dilation of tubular lumen. Furthermore, the product of the Glo damage score and TI damage score was defined as “kidney damage score.” The median of the scores for each dog was expressed as the values of each group.

For LMD, deparaffinized sections were stained with toluidine blue. LMD was performed using a MicroBeam Rel.4.2 (Carl Zeiss; Oberkochen, Germany) on 200–400 Glo and TI sections with an area equal to that of the total area from which samples were collected. Total RNA from LMD samples was isolated using a miRNeasy Micro Kit (Qiagen).

### miRNA analysis

miRNA levels in UExo-rich fractions and LMD samples were determined using a TaqMan MicroRNA RT Kit (ThermoFisher Scientific). Quantitative PCR analysis was performed using each miRNA-specific TaqMan primer and TaqMan Universal PCR Master Mix (ThermoFisher Scientific) with an MX3000P system (Agilent). Mimic miRNAs (AccuTarget; Bioneer, Daejeon, Republic of Korea) were used to draw standard curves, and the net level of miRNA was calculated by numerical formula.

### Statistical analyses

Results are expressed as the median or mean ± standard errors (SE). The Mann–Whitney *U* test was used to compare two groups (*P* < 0.05). Kruskal-Wallis test was used for comparing over three populations or time points, and multiple comparisons were performed using Scheffé's method when a significant difference was observed (*P* < 0.05). Spearman’s correlation test (*P* < 0.05) was used to analyze the correlation between two parameters.

## Additional Information

**How to cite this article:** Ichii, O. *et al*. Urinary exosome-derived microRNAs reflecting the changes of renal function and histopathology in dogs. *Sci. Rep.* **7**, 40340; doi: 10.1038/srep40340 (2017).

**Publisher's note:** Springer Nature remains neutral with regard to jurisdictional claims in published maps and institutional affiliations.

## Supplementary Material

Supplementary Information

## Figures and Tables

**Table 1:** List of differentially or similarly expressed UExo-derived miRNAs in KD compared to HC dogs.

	Accession	Fold change (KD vs. HC)	HC (raw)	KD (raw)	HC (normalized)	KD (normalized)	Reference
Differentially expressed UExo-derived miRNAs	miR-3107-5p^a^	18.5	153	3674	6.7	10.9	MI0014103
	miR-486a-5p	13.91	142	2564	6.6	10.4	MI0003493
	miR-21a-5p	4.09	211	1120	7.2	9.2	MI0000569
	miR-10a-5p	2.31	3665	10999	13	14.1	MI0000685
	miR-10b-5p	2.16	11980	33624	11.3	12.5	MI0000221
Similarly expressed UExo-derived miRNAs	miR-192-5p	1.87	304	737	7.71	8.61	MI0000551
	miR-22-3p	1.81	305	717	7.72	8.57	MI0000570
	miR-30a-5p	1.78	756	1749	9.03	9.86	MI0000144
	miR-191-5p	1.65	307	658	7.72	8.45	MI0000233

A total of 2,035 miRNAs were annotated. These genes showed 100 > read number and a 2-fold change in the kidney diseased group (KD) when compared to the healthy kidney control group (HC). a: This miRNA is updated as miR-486b-5p. miR-486a and miR-486b have the same mature sequence “uccuguacugagcugccccgag” in the mouse data base.

A total of 2,035 miRNAs were annotated. These genes showed 200 > read number and within a 2-fold change in the KD group compared to the HC group.

**Table 2:** Correlations between UExo-derived miRNA levels or ratio and clinical parameters in dogs.

Parameters		Age	BUN	Serum Cr	Urinary Cr
UExo-derived miRNA levels	miR-26a	−0.18	−0.397**	−0.303**	0.425**
	miR-146a	0.251*	0.130	0.115	−0.037
	miR-486	−0.111	−0.149	−0.159	0.314**
	miR-21a	0.028	0.213	0.238*	−0.200
	miR-10a	−0.06	−0.203	−0.221*	0.271*
	miR-10b	−0.161	−0.344**	−0.311**	0.428**
	miR191	−0.134	−0.218	−0.234*	0.270*
UExo-derived miRNA ratio	miR-146a/miR-26a	0.411**	0.362**	0.294**	−0.302**
	miR-486/miR-26a	0.095	0.147	0.030	−0.080
	miR-191/miR-26a	0.059	0.051	−0.041	−0.040
	miR-21a/miR-26a	0.134	0.489**	0.429**	−0.498**
	miR-10a/miR-26a	0.101	0.149	0.026	−0.06
	miR-10b/miR-26a	−0.034	0.05	−0.048	0.073
	miR-26a/miR-191	−0.016	−0.132	−0.016	−0.005
	miR-146a/miR-191	0.189	0.068	0.188	−0.222
	miR-486/miR-191	0.060	0.049	0.041	−0.071
	miR-21a/miR-191	0.251*	0.517**	0.512**	−0.412**
	miR-10a/miR-191	0.103	0.068	0.072	−0.058
	miR-10b/miR-191	−0.015	0.001	0.063	0.051

n = 75–84. Spearman’s rank correlation coefficient. *P < 0.05. **P < 0.01. BUN: blood urea nitrogen. Cr: creatinine.

**Table 3:** Correlations between KD to HC miRNA expression ratio and clinical parameters.

KD/HC ratio		Age	BUN	Serum Cr	Glo score	TI score	Disease score
Glo	miR-26a	−0.078	−0.627*	−0.823**	−0.585*	−0.393	−0.511
	miR-146a	−0.175	−0.482	−0.662*	−0.529	−0.273	−0.411
	miR-486	−0.418	−0.191	−0.395	−0.520	−0.689**	−0.600*
	miR-21a	−0.143	−0.345	−0.497	0.025	−0.036	−0.033
	miR-10a	−0.244	−0.755**	−0.795**	−0.772**	−0.675*	−0.739**
	miR-10b	−0.230	−0.609*	−0.681*	−0.806**	−0.736**	−0.778**
TI	miR-26a	0.005	−0.364	−0.584	−0.266	−0.301	−0.306
	miR-146a	0.345	−0.218	−0.290	0.140	0.368	0.228
	miR-486	0.046	−0.255	−0.354	−0.422	−0.458	−0.439
	miR-21a	0.212	0.336	0.106	0.607*	0.505	0.556*
	miR-10a	0.175	−0.409	−0.317	−0.104	−0.085	−0.150
	miR-10b	−0.189	−0.709*	−0.740**	−0.657*	−0.773**	−0.756**

n = 11–13. Glo: glomerular lesion. TI: tubulointerstitial lesion. BUN: blood urea nitrogen. Cr: creatinine. Expression ratio of the diseased group to the healthy group is analyzed. Spearman’s rank correlation coefficient. *P < 0.05. **P < 0.01. Disease score means histopathological index, including both Glo and TI.

**Table 4:** Correlations between Glo to TI miRNA expression ratio and clinical parameters.

Glo/TI ratio	Age	BUN	Serum Cr	Glo score	TI score	Disease score
miR-26a	−0.428	−0.400	−0.455	−0.702**	−0.477	−0.578*
miR-146a	−0.497	−0.327	−0.483	−0.607*	−0.580*	−0.589*
miR-486	−0.575	0.173	0.018	−0.196	−0.318	−0.256
miR-21a	−0.326	−0.500	−0.446	−0.534	−0.469	−0.500
miR-10a	−0.267	−0.573	−0.685*	−0.621*	−0.527	−0.572*
miR-10b	−0.621*	−0.173	−0.221	−0.448	−0.262	−0.339

n = 11–13. Glo: glomerular lesion. TI: tubulointerstitial lesion. BUN: blood urea nitrogen. Cr: creatinine. Expression ratio of Glo to TI is analyzed. Spearman’s rank correlation coefficient. *P < 0.05. **P < 0.01. Disease score means histopathological index, including both Glo and TI.
