# Supplementary material for: Urinary exosome-derived microRNAs reflecting the changes of renal function and histopathology in dogs
Source: Sci Rep. 2017 Jan 11;7:40340. doi: 10.1038/srep40340 (PMC5225487; doi:10.1038/srep40340)
Supplement: Supplementary Information [file srep40340-s1.pdf]

# Urinary exosome-derived microRNAs reflecting the changes of renal function and histopathology in dogs

**Osamu Ichii[1,*], Hiroshi Ohta[2], Taro Horino[3], Teppei Nakamura[1,4], Marina Hosotani[1], Tatsuya Mizoguchi[1], Keitaro Morishita[5], Kensuke Nakamura[5], Yuki Hoshino[5], Satoshi Takagi[5], Noboru Sasaki[2], Mitsuyoshi Takiguchi[2], Ryo Sato[6], Kazuhisa Oyamada[6], Yasuhiro Kon[1]**

[1]Laboratory of Anatomy, Department of Biomedical Sciences, Graduate School of Veterinary Medicine, Hokkaido University

[2]Laboratory of Veterinary Internal Medicine, Department of Veterinary Clinical Sciences, Graduate School of Veterinary Medicine, Hokkaido University

[3]Department of Endocrinology, Metabolism and Nephrology, Kochi University School of Medicine

[4]Section of Biological Safety Research, Chitose Laboratory, Japan Food Research Laboratories

[5]Veterinary Teaching Hospital, Graduate School of Veterinary Medicine, Hokkaido University

[6]Matsubara Animal Hospital

***Corresponding author**

Osamu Ichii, D.V.M., Ph.D.

Laboratory of Anatomy, Department of Biomedical Sciences, Graduate School of Veterinary Medicine, Hokkaido University, Kita 18-Nishi 9, Kita-ku, 060-0818 Sapporo, JAPAN. Tel & Fax: +81-11-706-5189.

E-mail: ichi-o@vetmed.hokudai.ac.jp

**Supplemental Fig. 1. Summary of analyzed dog.**

Values show the number of animals.

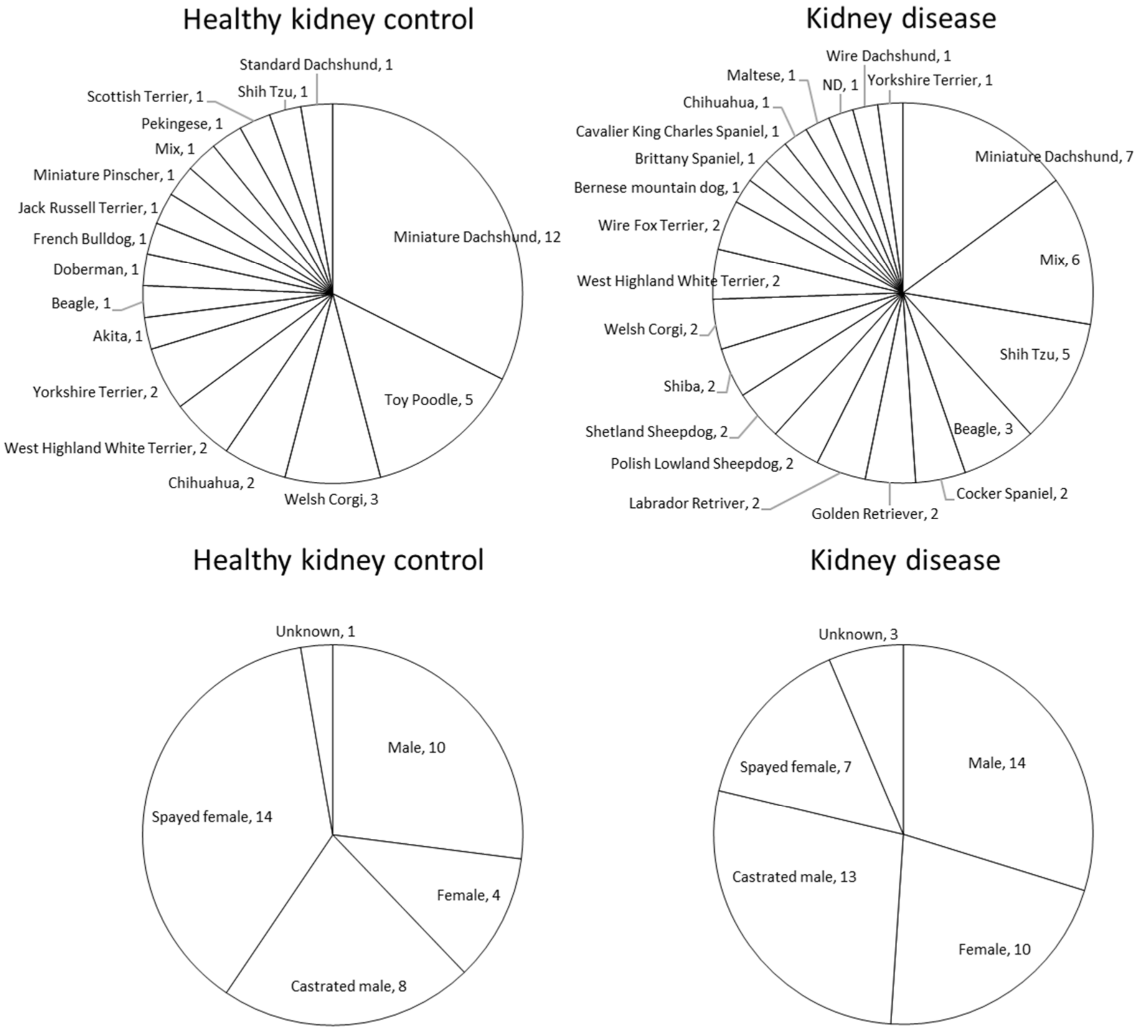

**Supplemental Table1. Summary of the analyzed dogs used for urine analysis.**

| Healthy control (urine) | | | Kidney disease (urine) | | |
|---|---|---|---|---|---|
| Species | Sex | Age | Species | Sex | Age |
| Akita | F* | 5 | Beagle | M | 15 |
| Beagle | F* | 11 | Beagle | M* | 14 |
| Chihuahua | M | 7 | Beagle | M | 14 |
| Chihuahua | M | 11 | Bernese mountain dog | M | 11 |
| Doberman | M | 2 | Brittany Spaniel | F | 13 |
| French Bulldog | F* | 6 | Cavalier King Charles Spaniel | M* | 9 |
| Jack Russell Terrier | F* | 9 | Chihuahua | F | 9 |
| Miniature Dachshund | M* | 11 | Cocker Spaniel | M | 13 |
| Miniature Dachshund | M | 13 | Cocker Spaniel | M | 9 |
| Miniature Dachshund | F | 6 | Golden Retriever | F | 11 |
| Miniature Dachshund | M | 12 | Golden Retriever | M | 8 |
| Miniature Dachshund | M* | 11 | Labrador Retriever | M | 7 |
| Miniature Dachshund | F* | 12 | Labrador Retriever | F | 15 |
| Miniature Dachshund | M* | 11 | Maltese | M* | 9 |
| Miniature Dachshund | F | 13 | Miniature Dachshund | F | 17 |
| Miniature Dachshund | F | 11 | Miniature Dachshund | M | 10 |
| Miniature Dachshund | Un | 8 | Miniature Dachshund | F | 13 |
| Miniature Dachshund | M* | 9 | Miniature Dachshund | F* | 13 |
| Miniature Dachshund | M* | 9 | Miniature Dachshund | F | 15 |
| Miniature Pinscher | M* | 10 | Miniature Dachshund | M | 8 |
| Mix | F* | 11 | Miniature Dachshund | M | 11 |
| Pekingese | M | 1 | Mix | F* | 11 |
| Scottish Terrier | F* | 11 | Mix | M* | 9 |
| Shih Tzu | F* | 17 | Mix | F | 15 |
| Standard Dachshund | M* | 10 | Mix | F* | 15 |
| Toy Poodle | F* | 6 | Mix | F* | 10 |
| Toy Poodle | M | 5 | Mix | M* | 8 |
| Toy Poodle | M | 7 | ND | ND | 15 |
| Toy Poodle | F | 13 | Polish Lowland Sheepdog | M* | 12 |
| Toy Poodle | F* | 7 | Polish Lowland Sheepdog | M* | 13 |
| Welsh Corgi | F* | 11 | Shetland Sheepdog | F | 10 |
| Welsh Corgi | M | 2 | Shetland Sheepdog | M* | 16 |
| Welsh Corgi | F* | 10 | Shiba | F* | 13 |
| West Highland White Terrier | F* | 11 | Shiba | F | 1 |
| West Highland White Terrier | F* | 11 | Shih Tzu | M | 16 |
| Yorkshire Terrier | M* | 8 | Shih Tzu | M | 15 |
| Yorkshire Terrier | M | 11 | Shih Tzu | F* | 12 |
| | | | Shih Tzu | M | 12 |
| | | | Shih Tzu | ND | 12 |
| | | | Welsh Corgi | M* | 13 |
| | | | Welsh Corgi | M | 13 |
| | | | West Highland White Terrier | ND | 14 |
| | | | West Highland White Terrier | F* | 12 |
| | | | Wire Dachshund | M* | 12 |
| | | | Wire Fox Terrier | M* | 12 |
| | | | Wire Fox Terrier | M* | 12 |
| | | | Yorkshire Terrier | M* | 12 |

M; Male. F: female. M*: Castrated male. F*: Spayed female. ND: not determined.

**Supplemental Table 2. Summary of analyzed dogs used for kidney analysis.**

| Healthy kidney control (Tissue) | | | Kidney disease (Tissue) | | |
|---|---|---|---|---|---|
| **Species** | **Sex** | **Age** | **Species** | **Sex** | **Age** |
| Beagle | F | 14 | Beagle | ND | ND |
| Beagle | M | 11 | Maltese | M* | 13 |
| Beagle | M | 11 | Miniature Dachshund | F | 8 |
| Miniature Schnauzer | M | 11 | Mix | F | 16 |
| Shih Tzu | M* | 3 | Pomeranian | M* | 9 |
| ND | ND | ND | Welsh corgi | M | 12 |
| | | | Yorkshire Terrier | F* | 8 |

M; Male. F: female. M*: Castrated male. F*: Spayed female. ND: not determined.
